# Innovationen in der Versorgung von Schwangerschaftsabbrüchen während der COVID-19-Pandemie in Deutschland, Frankreich und Großbritannien: Aufrechterhaltung des Zugangs und Aufbau von Resilienz

**DOI:** 10.1007/s00103-024-03995-2

**Published:** 2024-12-11

**Authors:** Céline Miani, Jana Niemann

**Affiliations:** 1 AG Epidemiologie und International Public Health, Fakultät für Gesundheitswissenschaften, Universität Bielefeld, Universität Str. 25, 33615 Bielefeld, Deutschland; 2 Institut für Medizinische Soziologie (IMS), Profilzentrum für Gesundheitswissenschaften, Martin-Luther-Universität Halle-Wittenberg, Halle (Saale), Deutschland

**Keywords:** Schwangerschaftsabbruch, Europa, COVID-19-Pandemie, Innovation, Gesundheitssystem, Abortion, Europe, COVID-19 pandemic, Innovation, Health systems

## Abstract

Die COVID-19-Pandemie hatte drastische Auswirkungen auf die Gesundheitssysteme vieler Länder. Zur Aufrechterhaltung der Versorgung mussten umgehend Lösungen gefunden werden, wobei sich auch das Potenzial ergab, Innovationen zu beschleunigen. So wurde beispielsweise der Zugang zu Schwangerschaftsabbrüchen während der Pandemie durch „Telehealth für frühen medikamentösen Schwangerschaftsabbruch“ (TEMA) verstärkt genutzt.

In diesem narrativen Übersichtsartikel werden Deutschland, Frankreich und Großbritannien in Hinblick auf die Entwicklung der Anzahl, Methoden und Settings von Schwangerschaftsabbrüchen in den Jahren 2018–2023 verglichen. Veränderungen im Versorgungsangebot und in der Gesetzgebung während der Pandemie werden dargestellt und die Unterschiede zwischen den Ländern sowie die verschiedenen Innovationsfaktoren diskutiert. Dazu wurden nationale Statistiken analysiert und es fand eine Literatur- und Onlinerecherche (Rapid-Review) statt.

In den 3 Ländern zeigen sich unterschiedliche Abbruchraten und Anteile medikamentöser Abbrüche sowie Auswirkungen der Pandemie; ein Anstieg von Telehealth ist aus einigen Quellen ersichtlich. In Frankreich und Großbritannien, wo medikamentöser Schwangerschaftsabbruch die Hauptmethode des Abbruchs ist, waren Dienstleistungen im Zusammenhang mit Schwangerschaftsabbrüchen schon vor der Pandemie leichter zugänglich. Während der Pandemie wurde hier TEMA, inkl. Versenden von Medikamenten, offiziell eingeführt. In Deutschland wurde TEMA erstmals durch Nichtregierungsorganisationen angeboten. Nachhaltigkeit und Verbreitung der Neuerungen bleiben fragil, insbesondere in Deutschland, wo der disruptive Ansatz einiger Organisationen noch keinen Eingang in die allgemeinen Gesundheitsdienste gefunden hat.

## Abstract (en)

The COVID-19 pandemic has had a drastic impact on healthcare systems. They had to react, adapt and innovate in order to build resilience, that is maintain healthcare access and health equity. For example, access to abortion services during the pandemic was increasingly facilitated through “Telehealth for Early Medical Abortion” (TEMA).

This narrative review article compares Germany, France and Great Britain in terms of abortion numbers, methods and settings from 2018 to 2023. Changes in the availability of services and legislation during the pandemic are presented, and the differences between the countries, as well as various innovation factors, are discussed. We used national statistics and conducted a literature and online search (Rapid Review).

In the three countries, there are differences in abortion rates, the share of medical abortions and the impact of the pandemic. In France and Great Britain, where medical abortion is the main method of abortion and where abortion care was more accessible before the pandemic than in Germany, a series of innovations were officially introduced to facilitate access during the pandemic. They included teleconsultations and the mailing of abortion medication. Most changes have been sustained since then, contributing to addressing historic and systemic health inequities in terms of access. In Germany, innovations during the pandemic have been crafted mainly by civil society organisations, offering for the first time teleabortion services.

The COVID-19 pandemic provoked or accelerated innovation in terms of abortion care in France, Germany and Great Britain. The sustainability and scaling-up of those innovations remain fragile, especially in Germany, where the disruptive approach of civil society organisations has not yet found its way into mainstream healthcare services.

## Einleitung

Schocks und plötzlich auftretende Krisen, welche eine kurze bis mittelfristige Entwicklung haben, stellen eine Bedrohung für Gesundheitssysteme dar [1]. Sie veranlassen Gesundheitssysteme dazu, zu reagieren, sich anzupassen, sich zu verändern und Innovationen zu entwickeln, um deren Resilienz zu stärken und so den Zugang zur Gesundheitsversorgung und die gesundheitliche Chancengleichheit zu erhalten. Die COVID-19-Pandemie war ein „System-Schock“ für die Gesundheitssysteme [2, 3] und veränderte die Bereitstellung und Erbringung von Gesundheitsleistungen in vielerlei Hinsicht (z. B. Personalmangel, Kontaktbeschränkungen im Alltag und in der Gesundheitsversorgung, Zunahme von häuslichen Betreuungsaufgaben aufgrund von Kita- und Schulschließungen und der daraus resultierende Mangel an Privatsphäre und Zeit für persönliche Termine).

In der Pandemie wurde auch der Zugang zu Schwangerschaftsabbrüchen zu einer besonderen Herausforderung, da diese nur in einem kurzen Zeitraum zu Beginn der Schwangerschaft durchgeführt werden sollten, oft schwer zugänglich sind und viele Frauen weite Wege zurücklegen müssen, um Hilfe zu erhalten [4]. Darüber hinaus sind sie häufig mit sozialer Stigmatisierung verbunden und erfordern in der Regel mehrere persönliche Termine [5]. In Deutschland beispielsweise sind oft bis zu 3 verschiedene Leistungserbringende (z. B. beratende Organisation, Gynäkolog*in und Gynäkolog*in, der/die Abbrüche durchführt) an einem Schwangerschaftsabbruch beteiligt, der je nach Verfahren aus 3–4 Terminen einschließlich eines Beratungstermins besteht.

Zu Beginn der COVID-19-Pandemie warnten Anbietende von Schwangerschaftsabbrüchen und Befürworter*innen sexueller und reproduktiver Rechte vor den Gefahren, die von der Pandemie ausgingen (z. B. [6, 7]): Das Prozedere von Schwangerschaftsabbrüchen müsste angepasst werden, wenn der Zugang aufrechterhalten werden sollte. Der Zugang zum Schwangerschaftsabbruch ist ein zentraler Aspekt der sexuellen und reproduktiven Rechte [8] und die Versorgung mit Schwangerschaftsabbrüchen ist daher eine wesentliche Gesundheitsdienstleistung [9]. Gesundheitssysteme sind aber relativ langsam, wenn es um Innovation geht. Innovation wird verstanden als „jedes Produkt, jede Technologie oder Dienstleistung, die für [ein Gesundheitssystem] neu ist oder auf eine Weise angewendet wird, die für [das Gesundheitssystem] neu ist, und die darauf abzielt, eine erschwingliche und qualitativ hochwertige Versorgung bereitzustellen“ [10].^1^ Es erfordert per se eine besonders fruchtbare Kombination von Umständen, die zur richtigen Zeit am richtigen Ort zusammentreffen, um Veränderungen anzunehmen und auf den Weg zu bringen. Der Raum für Innovationen in Gesundheitssystemen wird durch Hindernisse wie Verwaltungsprozesse, (fehlende) Finanzierung, Aversionen gegen Risiken, Sektorisierung und Kommunikationsprobleme eingeschränkt [10].

In den letzten Jahren hat sich gezeigt, dass hinsichtlich Innovationen in der Versorgung mit Schwangerschaftsabbrüchen das Potenzial für Weiterentwicklung eher in innovativen Versorgungsmodellen im Bereich des (medikamentösen) Schwangerschaftsabbruchs liegt [11, 12] als in der Entwicklung neuer Methoden oder Produkte. Im Vergleich zu Produktinnovationen (z. B. die Einführung eines neuen Medikaments), die mit hohen Investitionen und einem hohen Risikoprofil „boomen oder scheitern“ können, sind Innovationen in der Bereitstellung von Versorgung einfacher und schneller zu entwickeln bzw. umzusetzen [13]. Auch als eine Antwort auf die spezifischen Zwänge, die sich aus den Maßnahmen zur Eindämmung von SARS-CoV‑2 im Gesundheitswesen ergaben, haben sich Veränderungen in der Versorgung mit „frühem medikamentösem Schwangerschaftsabbruch“ (Early Medical Abortion: EMA) und insbesondere Innovationen im Bereich der „Telehealth für den frühen medikamentösen Schwangerschaftsabbruch“ (Telehealth for Early Medical Abortion: TEMA) ergeben [14]. Telemedizin oder Telehealth^2^ für den medikamentösen Schwangerschaftsabbruch bedeutet Onlineberatung der Schwangeren, inklusive Zusendung von entsprechenden Medikamenten auf dem Postweg, vorausgesetzt, es besteht Zugang zu genauen Informationen (z. B. über mögliche Schmerzen oder Nebenwirkungen), zu qualitätsgesicherten Medikamenten und bei Bedarf zur Unterstützung durch geschultes Gesundheitspersonal. Es hat sich gezeigt, dass TEMA auch ohne persönlichen Termin sicher ist [15] und von den aufsuchenden Personen geschätzt wird [16, 17].

Im Jahr 2022 veröffentlichte die Weltgesundheitsorganisation (WHO) aktualisierte Leitlinien zum Schwangerschaftsabbruch und führte die Telemedizin für den medikamentösen Abbruch als eine Methode ein, die den Zugang zu sicheren und effektiven Schwangerschaftsabbrüchen verbessern könnte [18]. Sie wird bis zur 12. Schwangerschaftswoche empfohlen. Diese Leitlinien unterstützen die Arbeit von Netzwerken und Organisationen, die seit Jahren, manchmal seit Jahrzehnten, medikamentöse Schwangerschaftsabbrüche in Ländern anbieten, in denen Schwangerschaftsabbrüche illegal sind oder der Zugang zu Schwangerschaftsabbrüchen eingeschränkt ist [19].

Da die COVID-19-Pandemie auf viele Länder starke Auswirkungen hatte, bietet sich die Gelegenheit, Innovationen in verschiedenen Gesundheitssystemen zu untersuchen und zu sehen, wie dort verschiedene Ebenen zusammenarbeiten, um Veränderungen zu bewirken und aufrechtzuerhalten. Hodgins und Kolleg*innen [2] entwickelten das „COVID-19 System Shock Framework“ (CSSF) auf der Grundlage von Hanefeld et al. [1] als „effektives Werkzeug zur Darstellung der komplexen, heterogenen Innovationen und Veränderungen, die als Reaktion auf die COVID-19-Pandemie im australischen Gesundheitssystem und darüber hinaus auftraten“. Das Modell berücksichtigt die Rolle der Gesundheitsdienste, des Gesundheitspersonals, der Informationssysteme, der Produkte und Technologien sowie der Finanzierung und des Finanzwesens in Verbindung mit dem Einfluss der Werte des Gesundheitssystems und der Politik und Verwaltung. Wir schlagen vor, die Innovation im Bereich des Schwangerschaftsabbruchs vor dem Hintergrund dieses Modells zu untersuchen. Wir haben 3 europäische Länder dafür ausgewählt, die vor der Pandemie unterschiedliche Versorgungstrukturen für den Schwangerschaftsabbruch hatten: Deutschland, Frankreich und Großbritannien. Durch diesen Vergleich sollen die zentralen Werte, die der Innovation zugrunde liegen, die politischen Steuerungsprinzipien, die den Wandel ermöglichen, und die Mittel, die zum Aufbau von Systemresilienz des Gesundheitssystems eingesetzt werden, herausgearbeitet werden.

Alle 3 Länder verfügen über hochentwickelte und leistungsfähige Gesundheitssysteme, gelten in Bezug auf die Gesetzgebung zum Schwangerschaftsabbruch als relativ liberal [20] und ermöglichen Schwangeren den Zugang zu Abbruchsdiensten auch ohne medizinischen Grund [21]. Alle 3 Länder bieten sowohl operative als auch medikamentöse Methoden des Schwangerschaftsabbruchs an [21]. Sie unterscheiden sich jedoch in Bezug auf:

- die Zugänglichkeit der Dienste (hürdenreicher in Deutschland, aufgrund z. B. einer Pflichtberatung und Wartezeit zwischen Beratung und Schwangerschaftsabbruch),
- die Kosten des Schwangerschaftsabbruchs (in Frankreich und Großbritannien für die meisten kostenlos),
- das Personal, das den Schwangerschaftsabbruch durchführt (in Frankreich bieten Hebammen den Schwangerschaftsabbruch an, in Deutschland nicht),
- die Einrichtungen, in denen der Schwangerschaftsabbruch durchgeführt wird (z. B. Krankenhaus, Gesundheitszentrum, zu Hause) und
- sozialkulturelle und politische Umgangsweisen mit Schwangerschaftsabbrüchen.

Ziel dieser narrativen Übersicht ist es, darzustellen, ob, wie und warum Innovationen in der Versorgung mit Schwangerschaftsabbrüchen während der Pandemie in Deutschland, Frankreich und Großbritannien eingeführt wurden und wo und wie diese Innovationen nach der Pandemie beibehalten, modifiziert oder aufgegeben wurden.

## Methoden

Diese narrative Übersichtsarbeit zum internationalen Vergleich von Trends in der Versorgung und Gesetzgebung zum Schwangerschaftsabbruch basiert auf einem methodenübergreifenden Ansatz und einer Triangulation von Datenquellen. Zunächst wurden quantitative und deskriptive Daten der nationalen statistischen Ämter von Deutschland, Frankreich und Großbritannien recherchiert, um die Trends und die Anzahl der Schwangerschaftsabbrüche, die Abbruchsraten, die Abbruchsmethoden und die Abbruchskontexte im Zeitraum 2018–2023 zu vergleichen.

Danach haben wir Peer-Review-Literatur und graue Literatur in einem „Rapid-Review“ [22] ausgewertet, um die neuesten Entwicklungen in der Abbruchsversorgung und der Abbruchsgesetzgebung nachzuvollziehen. Für die Peer-Review-Literatur haben wir eine Suche in Pubmed durchgeführt, bei der wir die Namen der interessierenden Länder mit dem Wort „Abortion“ kombiniert haben (Originalsuche: Abortion AND (UK OR Wales OR England OR Scotland OR France OR Germany)). Diese Suche fand für den Zeitraum 01.01.2020 bis 24.04.2024 statt. Für die graue Literatur nutzten wir die erweiterten Einstellungen der Google-Suche und kombinierten wiederum die Ländernamen und die Wörter für Schwangerschaftsabbruch auf Deutsch, Französisch und Englisch (nämlich: IVG, interruption volontaire de grossesse, avortement, Abtreibung, Schwangerschaftsabbruch, abortion).

Darüber hinaus haben wir die Websites relevanter Organisationen nach Stellungnahmen, Positionspapieren, Berichten oder Leitlinien durchsucht, die seit Beginn der COVID-19-Pandemie veröffentlicht wurden. Dazu gehörten die WHO und nationale Websites, wie etwa die der französischen „Haute Autorité de santé“ (HAS) und der „Deutschen Gesellschaft für Gynäkologie und Geburtshilfe“ (DGGG) sowie des „National Health Service“ (NHS) und des „National Institute for Health and Care Excellence“ (NICE) in Großbritannien. Wir extrahierten relevante Daten zur Anzahl von Schwangerschaftsabbrüchen auch außerhalb der regulären Gesundheitsversorgung (z. B. Nichtregierungsorganisationen).

Schließlich haben wir unser wissenschaftliches Netzwerk genutzt und nationale Expert*innen kontaktiert, um ad hoc Informationen zu klärungsbedürftigen Punkten zu erhalten, die nicht aus den oben genannten Datenquellen gewonnen werden konnten.

## Ergebnisse

### Anzahl, Methoden und Settings der Schwangerschaftsabbrüche 2018–2023: nationale Zahlen

In Deutschland, Frankreich sowie zusammengenommen in Großbritannien zeichnen sich Unterschiede in den Abbruchraten und dem Anteil der medikamentösen Schwangerschaftsabbrüche ab (Tab. 1).

**Tab. 1:** Anzahl, Methoden und Settings der Schwangerschaftsabbrüche in Deutschland, Frankreich, England und Wales, Schottland (2018–2023). Quellen: Deutschland: [52]; Frankreich: [53] und [24]; England und Wales: [25]; Schottland: [26]

Land, Jahr	Gesamtzahl der Schwangerschaftsabbrüche	Medikamentöse Schwangerschaftsabbrüche (%)	Setting der Schwangerschaftsabbrüche (%)^a^		
*Deutschland*	*–*	*–*	*Krankenhaus*	*Gynäkolog. Praxis*	*–*
2018	100.986	27	20	80	–
2019	100.893	29	21	79	–
2020	99.948	33	19	81	–
2021	94.596	37	19	81	–
2022	103.927	39	17	83	–
2023	106.218	43	16	84	–
*Frankreich*	–	–	*Krankenhaus*	Praxis (Hausarzt*innen, Hebamme, Gynäkolog*innen)	*„Gesundheitszentrum“ (Centre de santé)*
2018	224.339	69	75	23	2
2019	232.244	70	74	24	3
2020	216.794	72	69	28	3
2021	217.633	76	65	31	4
2022	234.353	78	62	34	4
2023^b^	–	–	–	–	–
*England und Wales*^c^	–	–	*Krankenhaus*	*Unabhängiger Anbieter unter NHS-Vertrag*^d^	*Privater Sektor*^e^
2018	200.608	71	26	72	2
2019	207.384	73	24	74	1
2020	209.917	85	22	77	1
2021	214.256	87	21	77	1
2022	251.377	86	19	80	2
2023^b^	–	–	–	–	–
*Schottland*^c^	–	–	*EMA in einer Praxis*^f^	*EMA teilweise in einer Praxis, teilweise zu Hause*	*EMA zu Hause*
2018	13.372	86	64	22	–
2019	13.659	88	52	36	–
2020	13.984	97	26	35	36
2021	13.937	99	18	28	53
2022	16.607	99	17	25	56
2023	18.207	98	17	24	58

*EMA* Early Medical Abortion

^a^Aufgrund von Rundungen kann es vorkommen, dass sich die Prozentsätze der Schwangerschaftsabbrüche nicht zu 100 % aufaddieren

^b^Daten ab 2023 waren für Frankreich sowie England und Wales zum Zeitpunkt der Recherche nicht verfügbar

^c^Die Daten für England und Wales und für Schottland werden getrennt erhoben

^d^Anbieter von Schwangerschaftsabbrüchen, die einen Vertrag mit dem nationalen Gesundheitsdienst (englisch: National Health Service, NHS) haben. Das bedeutet, dass ihre Leistungen über das nationale Versicherungssystem finanziert werden und für die Patienten kostenlos sind

^e^Anbieter außerhalb des nationalen Gesundheitssystems. Die Kosten für ihre Dienstleistungen müssen von der Patient*in selbst getragen werden, sie werden nicht von der gesetzlichen Krankenkasse übernommen

^f^Die Statistiken für Schottland sind die einzigen, die die Art des EMAs im Detail angeben, anstatt die Art des Anbieters

In Deutschland finden die meisten Schwangerschaftsabbrüche in gynäkologischen Praxen statt, wobei der operative Abbruch nach wie vor den Großteil der Abbrüche ausmacht. Mit rund 63 Schwangerschaftsabbrüchen je 10.000 Frauen^3^ im gebärfähigen Alter weist Deutschland die niedrigste Abbruchrate der 3 untersuchten Länder auf (2023; [23]). In Frankreich sind etwa 3/4 der Schwangerschaftsabbrüche medikamentöse Abbrüche und 1/3 davon wird außerhalb des Krankenhauses vorgenommen. Die Abbruchrate liegt bei 162 Schwangerschaftsabbrüchen pro 10.000 Frauen im gebärfähigen Alter (2022; [24]). In Großbritannien sind die meisten Schwangerschaftsabbrüche medikamentöse Abbrüche und werden von unabhängigen Einrichtungen im Rahmen von Verträgen mit dem National Health Service (NHS) durchgeführt. Die Abbruchrate liegt bei 206 Schwangerschaftsabbrüchen je 10.000 Frauen im gebärfähigen Alter (2022) in England und Wales [25] und bei 176 in Schottland (2023; [26]).

Seit 2018 ist in allen Ländern ein ähnlicher Trend zu mehr medikamentösen Schwangerschaftsabbrüchen und mehr Abbrüchen außerhalb von Krankenhäusern zu beobachten (Tab. 1). In Großbritannien ist ein besonders starker Anstieg (+12 Prozentpunkte in England und Wales, +9 Prozentpunkte in Schottland) des Anteils medikamentöser Abbrüche zwischen 2019 und 2020 zu verzeichnen. Dieser Trend begann bereits vor der Pandemie und setzt sich seitdem fort. Während der Pandemie ist für die Jahre 2020 und 2021 ein Rückgang der Zahl der Schwangerschaftsabbrüche in Deutschland und Frankreich zu beobachten. Dies könnte bis zu einem gewissen Grad mit einem eingeschränkten Zugang zusammenhängen, aber auch mit insgesamt weniger Schwangerschaften während der Pandemie, wie es für Frankreich vorgeschlagen wurde [27].

Es ist schwierig, sich einen Überblick über die Zahl der Schwangerschaftsabbrüche zu verschaffen, die in den Gesundheitseinrichtungen mithilfe von TEMA durchgeführt werden. Vereinzelte Quellen geben begrenzten Aufschluss über das Ausmaß, in dem Telehealth in den einzelnen Ländern während der Pandemie durchgeführt wurde:

Zwischen Dezember 2020 und Juni 2022 nahmen 134 Personen die TEMA-Dienste des Berliner Familienplanungszentrums BALANCE in Anspruch [28], um ihre Schwangerschaft zu beenden, es wurden keine weiteren deutschen Anbieter gefunden. In Frankreich ist die Entwicklung von TEMA im Gesundheitswesen anscheinend weiter fortgeschritten. Gibelin und Kollegen (2021), die eine Umfrage unter Ärzt*innen in Südfrankreich durchführten, stellten fest, dass 44,7 % der Befragten, die EMA anboten, im Jahr 2020 auch TEMA anbieten würden [29]. Jedoch fanden Mallaury et al. in einer quantitativen Studie aller Schwangerschaftsabbrüche in der französischen Region „Provence-Alpes-Côte d’Azur“ nur 96 TEMA unter den „EMA zu Hause“ (eigenständige Medikamenteneinnahme im häuslichen Umfeld; *n* = 8177; [30]). Dies steht im Einklang mit den Zahlen der nationalen Statistik (Direction de la recherche, des études, de l’évaluation et des statistiques), die 748 TEMA im Jahr 2020 und 971 TEMA im Jahr 2021 angibt [31]. In Großbritannien wurden nach Angaben von „MSI Reproductive Choices UK“, einem der größten unabhängigen Anbieter von Schwangerschaftsabbrüchen, seit der Einführung im März 2021 150.000 TEMA durchgeführt. Diese Zahl wurde von mehreren Berufsverbänden (z. B. British Medical Association [32]) aufgegriffen und in der Argumentation für die Beibehaltung von TEMA nach der Pandemie verwendet [33].

Zu diesen Zahlen könnten noch die TEMA-Zahlen außerhalb des formellen Sektors hinzugezählt werden. Zum Beispiel die Teleberatung der internationalen Organisation „Women on Web“ (WoW), deren Hauptaufgabe darin besteht, Schwangerschaftsabbrüche in Kontexten mit gesetzlich eingeschränktem Zugang zu ermöglichen: In Deutschland verzeichnete WoW 1090 Teleberatungen im Jahr 2019, 2285 im Jahr 2020 und 2041 im Jahr 2021 [34, 35], in Frankreich 809 Teleberatungen im Jahr 2020 [36].

### Veränderungen im Versorgungsangebot und in der Gesetzgebung während der Pandemie

Die Entwicklung der Zahlen und die großen Unterschiede in der Inanspruchnahme von TEMA lassen sich zum Teil durch die Entwicklung der Gesetzgebung und der Empfehlungen zum Schwangerschaftsabbruch und deren Umsetzung in den 3 Ländern erklären.

#### Deutschland

Vor der Pandemie war der medikamentöse Schwangerschaftsabbruch in Deutschland weniger verbreitet als der operative Abbruch und EMA zu Hause war nicht Teil des offiziellen Versorgungsangebotes. Weder EMA zu Hause noch TEMA wurden zu Beginn der COVID-19-Pandemie offiziell eingeführt, als andere Länder die Initiative ergriffen, TEMA einzurichten oder auszubauen, um den eingeschränkten Zugang zu regulärer persönlicher Versorgung zu kompensieren [37]. Eine Ausnahme bildeten die obligatorischen Beratungsgespräche durch unabhängige Organisationen (z. B. pro familia), die telefonisch oder online durchgeführt werden konnten [14, 38]. Diese Modalitäten sind jedoch nach wie vor ein seltenes Phänomen und nur ein kleiner Teil der Beratungsgespräche wird per Telefon und noch weniger per Video geführt [39]. Inzwischen wurden die EMA zu Hause und speziell die TEMA von der Deutschen Gesellschaft für Gynäkologie und Geburtshilfe (DGGG) als möglicher Weg zum Schwangerschaftsabbruch anerkannt [40], in Anlehnung an die aktualisierten Leitlinien der WHO [18] und an ein Modellprojekt, das vom Familienplanungszentrum BALANCE und dem Verein „Doctors for Choice Germany“ entwickelt wurde [35]. Dieses innovative Modellprojekt bot eine Version von TEMA an, die jedoch nicht vollständig digital war, da eine Bestätigung der Schwangerschaft durch Gynäkolog*innen nach wie vor erforderlich war, um die Dienstleistung in Anspruch nehmen zu können [41]. Auch die (zusätzlichen) Kosten dieses Angebotes könnten ein Hindernis für die Inanspruchnahme gewesen sein, da sie nicht von den gesetzlichen Krankenkassen übernommen werden.

#### Frankreich

Im April 2020 wurde in Frankreich die Möglichkeit einer EMA zu Hause von der 7. bis zur 9. Schwangerschaftswoche implementiert, um dem erschwerten Zugang zu Schwangerschaftsabbrüchen zu begegnen. Diese (dauerhafte) Änderung wurde von Familienplanungs- und feministischen Organisationen begrüßt, nicht aber von einigen Mitgliedern des nationalen Kollegiums der Gynäkologen (Collège National des gynécologues et obstétriciens; [42]). Gleichzeitig wurde auch die Telehealth für Konsultationen eingeführt und die Leistungserbringer konnten Rezepte an Apotheken senden, wo die Frauen sie direkt abholen konnten [43]. Einige finanzielle und organisatorische Parameter des Gesundheitssystems (isolierte Einzelpraxen, geringer Digitalisierungsgrad, Unbekanntheit neuer Methoden) schränkten jedoch die Wirkung dieser Maßnahme ein [43].

#### Großbritannien

In Großbritannien schließlich waren kurz vor Ausbruch der Pandemie bereits einige Änderungen in Bezug auf EMA in Vorbereitung: 2018/2019 wurde die Einnahme der zweiten Tablette zu Hause für EMA zugelassen. Im März 2020 wurde für England, Wales und Schottland die Entscheidung getroffen, TEMA durch Anbietende im Rahmen eines NHS-Vertrags zuzulassen [44]. Die TEMA-Dienste wurden zunächst befristet zugelassen, d. h. bis zum Abklingen der Pandemie. Die Debatte über die Nachhaltigkeit von TEMA nach der Pandemie endete im Jahr 2022, als die EMA zu Hause nach einer Abstimmung im Parlament gesetzlich verankert wurde [44]. Dieses Ergebnis entstand aus einer engen Zusammenarbeit zwischen den Akteur*innen (Leistungserbringenden, Wohltätigkeitsorganisationen und Nichtregierungsorganisationen), die von den Royal Colleges unterstützt wurde und eine starke Mobilisierung in den Medien und politischen Kreisen erreichte [45].

## Diskussion

**Abb. 1:**
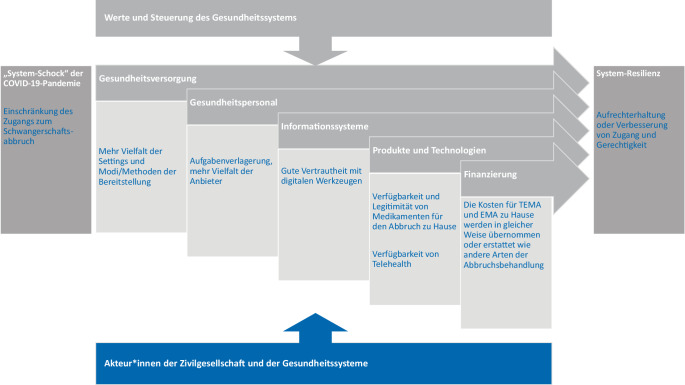
Framework für Innovationen in der Versorgung mit Schwangerschaftsabbrüchen nach dem System-Schock der COVID-19-Pandemie (nach Hodgins et al., [2]). Anmerkungen: Die Abbildung hebt die Akteur*innen und Bedingungen hervor, die Innovationen ermöglichen und den Zugang zu Schwangerschaftsabbrüchen erleichtern, von dem anfänglichen System-Schock bis hin zum Aufbau von Resilienz. *EMA* Early Medical Abortion, *TEMA* Telehealth for Early Medical Abortion. Quelle: eigene Darstellung

Wir haben nationale Trends und Initiativen in Bezug auf Schwangerschaftsabbrüche Versorgung in Deutschland, Frankreich und Großbritannien seit Beginn der COVID-19-Pandemie verglichen. Angesichts des System-Schocks der öffentlichen Gesundheit, der die traditionelle Art der Versorgung infrage stellte, mussten sich die Gesundheitssysteme in verschiedener Weise anpassen, um den Zugang zur Versorgung aufrechtzuerhalten [14, 37]. Dieser Vergleich von Zahlen, Methoden, Settings und Gesetzgebung zeigt eine Vielzahl von Faktoren, die bei der Entwicklung von Innovation eine Rolle spielen. Wir besprechen diese Faktoren im Folgenden und fassen sie in Abb. 1 zusammen, die an das Modell von Hodgins und Kolleg*innen [2] angelehnt ist. Sie stellt die wichtigsten Parameter der Innovation im Bereich des Schwangerschaftsabbruchs dar, von der Versorgungkrise im Gesundheitssystem (Health System Shock) bis hin zu einem resilienten Versorgungsangebot.

Wir stellen fest, dass die Weiterentwicklung der Versorgung mit Schwangerschaftsabbrüchen von den spezifischen Entwicklungen innerhalb der Länder abhängt. Selbst in Krisenzeiten gibt es eine *Pfadabhängigkeit* („path dependency“), die das Ausmaß der Umsetzung von Veränderungen beeinflusst. Innovation ist oft mehr eine Frage der Akzeptanz oder der Beschleunigung des Wandels [46] als ein Bruch oder ein völliger Paradigmenwechsel. So haben z. B. die Unterschiede im Umgang mit Schwangerschaftsabbrüchen in Frankreich, Deutschland und Großbritannien vor der Pandemie sicherlich eine Rolle bei der Anpassung des Systems während der Pandemie gespielt.

Es ist wichtig, darauf hinzuweisen, dass alle Innovationen im Bereich des Schwangerschaftsabbruchs mit dem medikamentösen Abbruch und insbesondere mit dem *EMA* und *TEMA* verbunden waren [14]. Dies erfordert einen rechtlichen Rahmen und Infrastrukturen, einschließlich *IT-Infrastrukturen*, die die Entwicklung solcher Dienste erleichtern. Die zentrale Rolle des medikamentösen Schwangerschaftsabbruchs bei der Aufrechterhaltung des Zugangs und der Gerechtigkeit ist nicht nur in Europa offensichtlich, sondern auch in den USA, wo die doppelte Krise der COVID-19-Pandemie und der Aufhebung des Urteils zum grundsätzlichen Recht auf Schwangerschaftsabbruch „Roe vs. Wade“ im Jahr 2022 zu einer tiefgreifenden Veränderung der Versorgung mit Schwangerschaftsabbrüchen geführt haben. In 2023 wurden in den USA schätzungsweise 19 % der Abbrüche telemedizinisch durchgeführt [47]. Ursprünglich befürchteten Befürwortende und Forschende, dass diese beiden Ereignisse den Zugang zu Schwangerschaftsabbrüchen erschweren würden, doch tatsächlich haben Innovation und Anpassung dazu geführt, dass bestimmte Bevölkerungsgruppen nun besser versorgt werden [48]: Die Ausweitung und Legitimierung von TEMA, einschließlich der Bestimmungen für eine gerechte Finanzierung, verbessern den Zugang insbesondere für die am stärksten gefährdeten oder marginalisierten Bevölkerungsgruppen, die zuvor aufgrund von Isolation, Kosten, Diskriminierung oder Stigmatisierung Schwierigkeiten hatten, persönliche Dienste in Anspruch zu nehmen.

Im Mittelpunkt der Innovationen stehen häufig eine *dezentrale Versorgungsstruktur und die Verlagerung von Aufgaben an nichtärztliches medizinisches Personal* [49]: Zu wissen, wie weit Frankreich, Deutschland und Großbritannien in diesen Bereichen fortgeschritten waren, ist wichtig, um zu verstehen, wie sie auf die Pandemie reagiert haben. Die Verlagerung der Versorgung außerhalb des Krankenhauses und die Zulassung der Begleitung von Schwangerschaftsabbrüchen durch nichtärztliches medizinisches Personal (z. B. Hebammen oder Apotheker*innen) sind von zentraler Bedeutung. In dieser Hinsicht ist die kontrastierende Situation zwischen den 3 Ländern aufschlussreich: Das am stärksten „Arzt-zentrierte“ System in Deutschland war nicht in der Lage, sich in dem Maße und mit der Schnelligkeit in Hinblick auf TEMA zu entwickeln wie die beiden anderen. Die Verlagerung von Aufgaben durch die historisch bedingte Einbindung von Hebammen und Dienstleistenden des dritten Sektors in Frankreich und noch stärker in Großbritannien dürfte die Innovation in der Gesundheitsversorgung gefördert haben.

Auch aus der Zusammenarbeit, den Synergien und der Mobilisierung der Akteur*innen im Bereich Versorgung mit Schwangerschaftsabbrüchen können sich wichtige Impulse für deren Weiterentwicklung ergeben. So haben beispielsweise in Großbritannien die Dominanz von Nichtregierungsorganisationen im Dienstleistungssektor bei der Bereitstellung von Versorgungsangeboten und das Engagement der Zivilgesellschaft bei der Gestaltung und Politisierung von Gesundheitsfragen die landesweite Etablierung von TEMA vorangebracht [32]. Diese Schlüsseldimension wird im Modell von Hodgins et al. nicht vollständig abgebildet, wo politische Steuerung und Werte nur Teilaspekte der Zivilgesellschaft und der Mobilisierung von Fachkräften abdecken können. Die Zivilgesellschaft und die Fachkräfte spielen eine wichtige Rolle, wenn es darum geht, Innovationen zu ermöglichen oder zu behindern, und bei Themen wie den sexuellen und reproduktiven Rechten muss ihr Einfluss von der Theorie bis zur Praxis berücksichtigt werden.

Die COVID-19-Pandemie hat dazu geführt, dass TEMA nicht mehr nur von *Organisationen im Kontext eingeschränkter Versorgungszugänge „am Rande des Gesundheitssystems“* (z. B. WoW) praktiziert wird, sondern u. a. in Frankreich und Großbritannien zu einem festen Bestandteil der offiziellen Gesundheitsdienste geworden ist. Die Arbeit dieser Organisationen hat bereits vor der Pandemie die Defizite der regulären Gesundheitsversorgung und geeignete Alternativen aufgezeigt und konnte nun einen positiven Beitrag zur Aufrechterhaltung der offiziellen Gesundheitsdienste während der Pandemie leisten [50]. Die störende und in gewisser Weise antagonistische Fähigkeit von Nichtregierungsorganisationen kann der Schlüssel für einen Systemwandel sein (hier: mehr Vielfalt im Angebot von Dienstleistungen rund um den Schwangerschaftsabbruch; [19]). Ihr Einfluss kann aber begrenzt sein, wenn rechtliche Restriktionen und Kriminalisierung von Schwangerschaftsabbrüchen aufrechterhalten bleiben.

2023 wurde in Deutschland eine Kommission „Reproduktive Selbstbestimmung und Fortpflanzungsmedizin“ eingesetzt, um Empfehlungen zur aktuellen Rechtslage zum Schwangerschaftsabbruch in Deutschland zu formulieren. Sie empfiehlt, Schwangerschaftsabbrüche innerhalb der ersten 12 Wochen zu legalisieren, da die derzeitige Regelung verfassungs-, völker- und europarechtlich nicht haltbar sei. Die Kommission fordert, dass frühe Schwangerschaftsabbrüche (bis zur 12. Woche) legal und straffrei sein sollen und sieht auch gesetzliche Spielräume für mittlere Phasen (bis zur 22. Woche; [51]). Eine vollständige Entkriminalisierung des Schwangerschaftsabbruchs könnte den Weg für mehr Synergien zwischen den verschiedenen Akteuren und für mehr Innovation in der Versorgung mit Schwangerschaftsabbrüchen ebnen.

## Fazit

Die COVID-19-Pandemie hat in Frankreich, Deutschland und Großbritannien Telehealth, inkl. Versand von Medikamenten per Post, als innovative Versorgungsangebote für Schwangerschaftsabbrüche befördert. Die Nachhaltigkeit und Verbreitung dieser Innovationen bleiben jedoch fragil, insbesondere in Deutschland, wo der disruptive Ansatz einiger professioneller und zivilgesellschaftlicher Organisationen noch keinen Eingang in die allgemeinen Gesundheitsdienste gefunden hat.

## Abkürzungen

EMA: Early Medical Abortion (früher medikamentöser Schwangerschaftsabbruch)
TEMA: Telehealth for Early Medical Abortion (früher medikamentöser Schwangerschaftsabbruch, unterstützt durch Telehealth)
WHO: Weltgesundheitsorganisation
WoW: Women on Web

## Footnotes

1 Wie diese Definition zeigt, bezieht sich Innovation nicht nur auf ein Produkt, sondern auch auf eine Dienstleistung und es geht nicht unbedingt darum, etwas Neues zu erfinden, sondern es auf eine neue Art und Weise oder in einem neuen Kontext umzusetzen.

2 Ein Begriff, der von feministischen Netzwerken und Online-Anbieter*innen von medikamentösen Schwangerschaftsabbrüchen bevorzugt wird, da sie den selbstbestimmten Abbruch fördern.

3 Wir erkennen an, dass Menschen unterschiedlicher Geschlechtsidentität Zugang zu Dienstleistungen im Zusammenhang mit Schwangerschaftsabbruch haben sollten und diese benötigen. Wir verwenden den Begriff „Frauen“, wenn wir andere Studien zitieren oder wenn wir uns in unserer Studie auf cisgeschlechtliche Frauen beziehen.
